# Prognostic significance of stress hyperglycemia ratio in coronary microvascular dysfunction among chronic coronary syndrome patients

**DOI:** 10.3389/fcvm.2026.1740092

**Published:** 2026-06-04

**Authors:** Jiasuer Alifu, Hailipai Mamuti, Yongqiang Luo, Chunyue Wang, Wen Zhang, Penglong Qi, Abdul-Quddus Mohammed, Lu Liu, Guoqing Yin, Xian Lv, Tingting Shi, Fuad A. Abdu, Wenliang Che

**Affiliations:** 1 Department of Cardiology, Shanghai Tenth People’s Hospital, Tongji University School of Medicine, Shanghai, China; 2 Department of Cardiology, Shanghai Tenth People’s Hospital Chongming Branch, Shanghai, China

**Keywords:** chronic coronary syndrome, coronary microvascular dysfunction, mortality, prognosis, stress hyperglycemia ratio

## Abstract

**Aims:**

Coronary microvascular dysfunction (CMD) is a crucial prognostic indicator in chronic coronary syndrome (CCS) patients. The stress hyperglycemia ratio (SHR), a novel biomarker of acute hyperglycemia, has demonstrated predictive value for adverse outcomes in various diseases. However, its association with CMD remains unclear. This study aims to evaluate the prognostic role of SHR in predicting CMD outcomes in CCS patients.

**Methods:**

CCS patients who underwent coronary angiography (CAG) were included. Microvascular function was assessed using coronary angiography-derived index of microcirculatory resistance (caIMR), with CMD defined as caIMR > 25U. Patients were divided into three groups based on SHR values: T1 (SHR < 0.73), T2 (SHR 0.73–0.80), and T3 (SHR≥0.80). The primary endpoint was major adverse cardiac events (MACE).

**Results:**

379 CCS patients were included, among which 196 were identified with CMD. The CMD group exhibited higher SHR levels. During a mean follow-up of 32.9 months, 53 MACE events occurred in CMD patients, with a higher incidence in the T3 group compared to the T1/T2 groups (37.681% vs. 16.667% vs. 26.230%, *P* = 0.023). SHR was identified as an independent predictor of CMD (OR 7.379, 95% CI: 1.305–41.725, *P* = 0.024). The T3 group demonstrated a 2.325-fold increased risk of MACE (HR 2.325, 95% CI: 1.228–5.033, *P* = 0.011), with a non-linear relationship observed between SHR and MACE (P for non-linear < 0.001).

**Conclusions:**

Elevated SHR is associated with adverse outcomes and predicts MACE in CMD patients with CCS. These results highlight the potential of SHR as a valuable tool for early risk stratification and prevention in CMD management.

**Trial Registration:**

ClinicalTrials.gov, NCT06698432 (Registration Date: November 19, 2024)

## Introduction

Coronary artery disease (CAD), a major cardiovascular condition caused by atherosclerotic narrowing or occlusion of coronary arteries, results in myocardial ischemia, hypoxia, or necrosis and remains one of the leading causes of morbidity and mortality globally (1, 2). Chronic coronary syndrome (CCS), the stable and progressive phase of CAD, is characterized by sustained clinical manifestations over time (3). Coronary microvascular dysfunction (CMD), a condition marked by structural and functional abnormalities in the coronary microcirculation, is frequently observed in clinical settings and is particularly associated with atherosclerosis, exacerbating myocardial ischemia in CCS patients (4–6). CMD is prevalent in various cardiovascular disorders, including heart failure with preserved ejection fraction (HFpEF), Takotsubo syndrome, acute coronary syndrome, myocardial infarction with non-obstructive coronary arteries (MINOCA), and CCS, playing a pivotal role in their pathophysiology and prognosis (4, 7–10). Substantial evidence underscores the high prevalence of CMD in CCS, linking it to disease progression and unfavorable outcomes (11, 12). Consequently, the diagnosis and identification of predictors of CMD are of critical importance for the effective management and prevention of adverse outcomes in CCS patients.

The molecular mechanisms of CMD remain unclear, however, oxidative stress and inflammatory responses are key contributors, impairing vessel dilation and increasing constriction (4, 13). Hyperglycemia plays a significant role in CMD by disrupting vascular regulation through altered cardiomyocyte oxygen demand (14, 15). Stress-induced hyperglycemia worsens CMD by promoting endothelial dysfunction and oxidative stress, leading to adverse outcomes (16). Acute hyperglycemia, often overlooked due to inadequate monitoring, may arise from chronic hyperglycemia or acute stress, causing transient blood glucose spikes at hospital admission. The stress hyperglycemia ratio (SHR), calculated as admission blood glucose (ABG) divided by estimated average chronic glycemia, is a novel marker reflecting acute hyperglycemia (17). SHR has shown prognostic value in conditions like MINOCA, HFpEF, and CAD, aiding survival prediction and risk stratification (18–20)**.** However, its prognostic significance in CMD among CCS patients remains unexplored, warranting further research.

Therefore, this study aims to evaluate the predictive value of SHR in CMD among CCS patients and to clarify its clinical relevance and significance, which remain insufficiently understood in this population.

## Materials and methods

### Study design and population

This single-center retrospective observational study was conducted at Shanghai Tenth People’s Hospital from June 2015 to June 2019 (NCT06698432). Patients aged over 18 years with suspected or established CCS, as defined by CCS guidelines, who underwent coronary angiography (CAG) were included (3). Exclusion criteria included a left ventricular ejection fraction (LVEF) below 35%, recent myocardial infarction (MI), severe hepatic or renal dysfunction, malignancy, prior coronary artery bypass graft surgery, pregnancy, incomplete SHR data, non-adherence to follow-up protocols, other life-threatening diseases affecting long-term survival, or suboptimal angiographic images due to vascular overlap, artery distortion, or low contrast opacification.

The study was approved by the Ethical Review Board of Shanghai Tenth People’s Hospital and adhered to the principles of the Helsinki Declaration (ethical number: 24K169), with written informed consent obtained from all participants.

### Data collection and definition

The medical records of all study participants were retrospectively reviewed to collect baseline demographic data and relevant clinical information. Demographic variables included age, sex, body mass index (BMI), heart rate, and blood pressure. Clinical details encompassed prior medical conditions such as diabetes mellitus, hyperlipidemia, hypertension, heart failure, atrial fibrillation, stroke, chronic kidney disease (CKD), smoking history, and history of percutaneous coronary intervention. Data on coronary angiography (CAG) findings, echocardiographic parameters, and medication history were also collected. Laboratory parameters were assessed using venous blood samples obtained after an overnight fast upon admission. These included white blood cell count, total cholesterol (TC), triglycerides (TG), low-density lipoprotein cholesterol (LDL-C), fasting blood glucose (FBG), hemoglobin A1c (HbA1c), cardiac troponin T (cTnT), hemoglobin (Hb), C-reactive protein (CRP), serum creatinine (SCr), aspartate aminotransferase (AST), total bilirubin, and albumin levels. Notably, analyses of blood glucose, TC, LDL-C, high-density lipoprotein cholesterol (HDL-C), and TG were performed using testing methods provided by Abbott Laboratories (Chicago, IL, USA).

The BMI was computed following the subsequent formula: BMI (kg/m²) is equal to the weight (kg) divided by the square of the height (m²). Hypertension was defined as systolic blood pressure (SBP) ≥ 140 mmHg or diastolic blood pressure (DBP) ≥ 90 mmHg either at rest or when the patient was undergoing antihypertensive treatments. The estimated glomerular filtration rate (eGFR) was calculated by employing the Modification of Diet in Renal Disease Study glomerular filtration rate equation. A diagnosis of diabetes was established based on the following criteria: (1) HbA1c ≥ 6.5%. (2) FBG ≥ 7.0 mmol/L (≥126 mg/dL); (3) Random plasma glucose ≥ 11.1 mmol/L (≥200 mg/dL); (4) Oral glucose tolerance test glucose level ≥ 11.1 mmol/L (200 mg/dL).

### Coronary microvascular function measurement

Coronary microvascular function was assessed in all participants using the caIMR in the Cathlab of Shanghai Tenth People’s Hospital. CAG was performed at the discretion of the operators, capturing multiple views at either 15 or 30 frames per second. The caIMR measurements were obtained using the Flash Angio system, which includes the Flash Angio console, software, and pressure transducer, all supplied by Rainmed Ltd., Suzhou, China. The estimation of caIMR followed a validated protocol utilizing computational pressure-flow dynamics in three sequential steps. First, a simulated three-dimensional reconstruction of the coronary arteries was created for the target vessels. Next, angio-Fractional Flow Reserve (angio-FFR) was estimated using computational pressure-flow dynamics, incorporating the estimated hyperemic aortic pressure (P'a) derived from mean aortic pressure. Finally, caIMR was calculated using the designated equation (21):

$$caIMR=Pd_{hyp}\frac{L}{K.V_{diastole}}$$

In the above calculation, *Pd _hyp_* is indicative of the mean pressure present at the distal position when maximal hyperemia occurs. Herein, *L* serves as a constant that represents the length stretching from the inlet to the distal position. The constant K has been assigned a value of precisely 2.1. Meanwhile, *V*_diastole_ denotes the mean flow velocity at the distal position during the diastolic phase. Additionally, *V_hyp_* is computed by multiplying *K* with *V_diastole_*, which represents the mean flow velocity at the distal position during the maximal hyperemia period.

The assessment of caIMR was carried out concerning at least one of the three coronary arteries, including the Left Anterior Descending Artery (LAD), the Left Circumflex Artery (LCX), and the Right Coronary Artery (RCA), for all the patients involved. Among these vessels, the epicardial artery with the highest caIMR value was selected for analysis. CMD was defined as a caIMR value > 25U, consistent with findings from previous studies (21).

### Calculation of SHR and grouping

The SHR was calculated using the formula: SHR = ABG (mmol/L)/ (estimated average glucose), where the estimated average glucose (mmol/L) was derived as 1.59 × HbA1c (%)—2.59 (22). Based on the calculated SHR values, patients were categorized into three groups: T1 group (SHR <0.73), T2 group (SHR: 0.73–0.80), and T3 group (SHR ≥0.80).

### Follow-up and endpoint of the study

The mean follow-up period of this study was 32.9 months, during which data were collected through telephone interviews, hospital records, and outpatient visits conducted by trained physicians at Shanghai Tenth People’s Hospital. MACE was defined as the primary clinical endpoint and included the following: (1) Cardiac death, resulting from malignant arrhythmia, acute MI, heart failure, or other cardiac conditions; (2) Ischemia-driven revascularization, performed due to recurrent angina or a positive cardiac ischemia test; (3) Nonfatal MI, identified by positive cardiac biomarkers along with typical symptoms of myocardial ischemia or dynamic electrocardiogram changes (23); (4) Heart failure, characterized by clinical symptoms such as dyspnea and fatigue, supported by echocardiographic evidence, elevated BNP/NT-proBNP levels, and cardiac function assessment (24); and (5) Nonfatal stroke, diagnosed as acute cerebral infarction based on clinical symptoms or imaging findings (25).

### Statistical analysis

Statistical analyses were performed using the Statistical Package for Social Sciences (SPSS) version 25, and figures were created with GraphPad version 9. Numeric variables were expressed as mean ± standard deviation and compared between groups using t-tests or ANOVAs. Categorical variables were presented as counts and percentages (%) and analyzed using Pearson chi-square or Fisher’s exact tests. Logistic regression analysis was conducted, incorporating significant clinical predictors from univariate models into multivariable models to assess the association between clinical risk factors and CMD. The Kaplan–Meier method was used to visualize time-related mortality, with differences evaluated through log-rank tests. Cox regression models were applied to calculate hazard ratios (HR) and 95% confidence intervals (CI) to identify predictors of clinical outcomes. In univariate analyses, covariates included all risk factors and angiographic parameters. Variables showing significant associations with the primary outcome in univariate analysis were subsequently included in multivariable models. To examine the non-linear relationship between SHR and MACE in CMD patients, restricted cubic spline regression was employed for HR analysis. Additionally, subgroup analyses were conducted to explore the association between SHR and stratified factors such as demographics and comorbidities, with interaction *P*-values used to assess heterogeneity between subgroups.

All analyses were carried out in a two-sided manner, and results were deemed statistically significant when the *P*-value was < 0.05.

## Results

### Baseline characteristics

**Figure 1:**
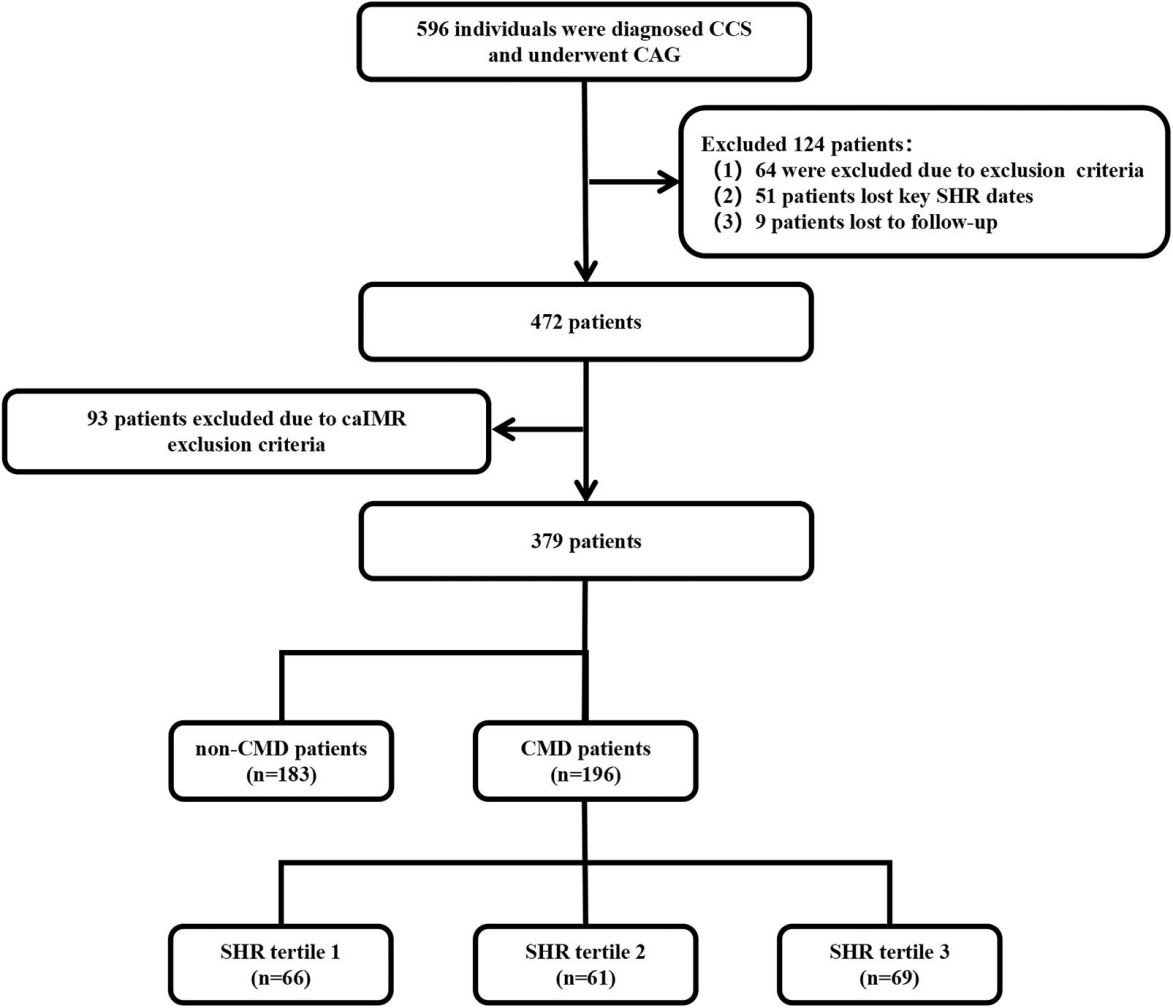
Recruitment Flow diagram of the study population. CCS, chronic coronary syndrome; CAG, coronary angiography; SHR, Stress hyperglycemia ratio; caIMR, coronary angiography-derived index of microcirculatory resistance, CMD, coronary microvascular dysfunction.

A total of 379 patients (57.653% were male, mean age was 63.464 ± 9.133) who underwent CAG and met the diagnostic criteria for CCS were included in the final analysis of the study, among which 196 were identified with CMD (Figure 1).

**Figure 2:**
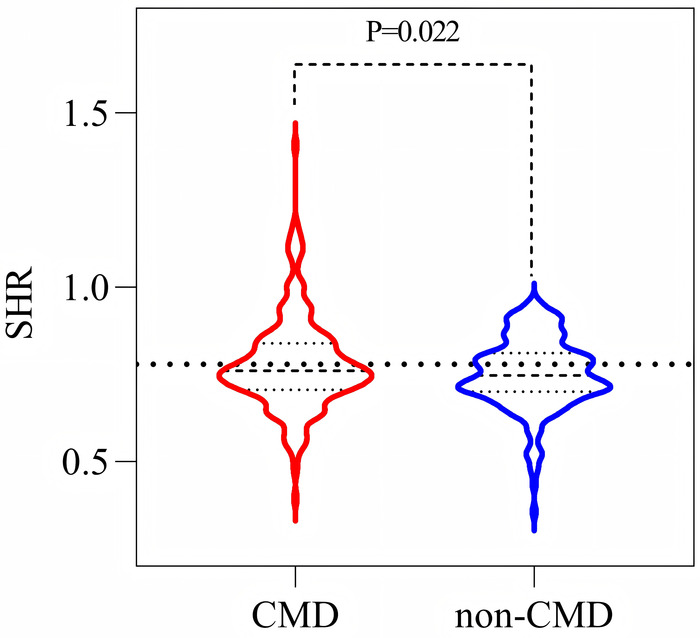
The distribution of SHR values at CMD and non-CMD patients. SHR, stress hyperglycemia ratio; CMD, coronary microvascular dysfunction.

Compared to those without CMD, patients with CMD had significantly higher SHR values (0.780 ± 0.136 vs. 0.751 ± 0.103; *P* = 0.002) (Figure 2). Additionally, CMD patients tended to have hyperlipidemia as well as exhibited elevated caIMR, caFFR, DBP, TG, LDL-C, TC, and FBG levels, while showing lower rates of prior PCI, 3-vessel disease, and clopidogrel use (Supplementary Table 1). According to SHR tertiles, CMD patients were divided into three groups (T1, T2, and T3). Those in the T3 group had significantly elevated laboratory parameters such as TC, TG, FBG, and albumin, while no notable differences were observed in demographic characteristics, comorbidities, or medication usage (Table 1).

**Table 1:** Clinical characteristics of the CMD patients stratified by SHR tertile.

		SHR tertile			
Variables	Total patient (*N* = 196)	T1 (*N* = 66)	T2 (*N* = 61)	T3(*N* = 69)	*P* value
General characteristics					
Age (years)	63.464 ± 9.133	64.667 ± 9.495	63.787 ± 9.381	62.029 ± 8.471	0.233
Male, *n* (%)	113 (57.653)	41 (62.121)	35 (57.377)	37 (53.623)	
SBP (mmHg)	133.15 ± 19.346	130.538 ± 20.634	135.746 ± 17.716	133.403 ± 19.390	0.325
DBP (mmHg)	80.136 ± 12.742	80.877 ± 11.516	80.475 ± 12.942	79.119 ± 13.782	0.711
Heart rate (bpm)	77.158 ± 11.400	75.561 ± 9.928	76.902 ± 11.727	78.913 ± 12.308	0.228
BMI (kg/m^2^)	25.631 ± 3.288	25.847 ± 3.237	25.551 ± 3.653	25.493 ± 3.031	0.803
Comorbidities					
Diabetes, *n* (%)	75 (38.265)	23 (34.848)	19 (31.148)	33 (47.826)	0.116
Hyperlipidemia, *n* (%)	75 (38.265)	23 (34.848)	25 (40.984)	27 (39.130)	0.794
Hypertension, *n* (%)	124 (63.265)	46 (69.697)	32 (52.459)	46 (66.667)	0.101
Heart failure, *n* (%)	2 (1.020)	0 (0.00)	2 (3.279)	0 (0.00)	0.096
Atrial fibrillation, *n* (%)	8 (4.082)	3 (4.545)	3 (4.918)	2 (2.899)	0.820
Stroke, *n* (%)	31 (15.816)	8 (12.121)	9 (14.754)	14 (20.290)	0.414
CKD, *n* (%)	18 (9.184)	9 (13.636)	6 (9.836)	3 (4.348)	0.171
Smoke, *n* (%)	42 (21.429)	18 (27.273)	10 (16.393)	14 (20.290)	0.315
PCI, *n* (%)	93 (47.449)	37 (56.061)	27 (44.262)	29 (42.029)	0.220
1-vessel disease	50 (25.510)	21 (31.818)	17 (27.869)	12 (17.391)	0.138
2-vessel disease	39 (19.898)	17 (25.758)	7 (11.475)	15 (21.739)	0.117
3-vessel disease	21 (10.714)	6 (9.091)	9 (14.754)	6 (8.696)	0.469
Coronary physiological values					
caFFR	0.949 ± 0.026	0.948 ± 0.028	0.948 ± 0.025	0.950 ± 0.026	0.872
caIMR	36.759 ± 9.895	37.412 ± 9.265	36.782 ± 10.615	36.114 ± 9.923	0.750
Laboratory parameters					
WBC (10^9/L)	6.499 ± 1.657	6.799 ± 1.587	6.092 ± 1.549	6.576 ± 1.760	0.059
TC (mmol/L)	3.935 ± 1.063	3.706 ± 0.928	3.917 ± 0.966	4.177 ± 1.220	0.037
HDL-C (mmol/L)	1.078 ± 0.285	1.039 ± 0.258	1.094 ± 0.290	1.102 ± 0.306	0.393
LDL-C (mmol/L)	2.175 ± 0.924	2.005 ± 0.889	2.171 ± 0.868	2.343 ± 0.987	0.109
TG (mmol/L)	1.912 ± 1.536	1.762 ± 1.106	1.604 ± 0.720	2.332 ± 2.210	0.018
SHR	0.780 ± 0.136	0.655 ± 0.068	0.760 ± 0.020	0.916 ± 0.117	<.001
FPG (mmol/L)	5.953 ± 1.982	5.012 ± 0.847	5.585 ± 1.073	7.177 ± 2.658	<0.001
Hb1Ac (%)	6.419 ± 1.104	6.495 ± 1.031	6.249 ± 0.878	6.496 ± 1.327	0.353
cTnT (ng/mL)	0.065 ± 0.640	0.014 ± 0.021	0.010 ± 0.006	0.164 ± 1.080	0.161
Hb (g/L)	134.792 ± 16.502	135.576 ± 13.110	132.056 ± 22.678	136.485 ± 12.281	0.282
CRP (mg/dL)	3.944 ± 7.609	3.842 ± 5.856	4.430 ± 11.691	3.609 ± 3.429	0.825
SCr (umol/L)	75.541 ± 18.951	78.668 ± 21.534	75.690 ± 17.374	72.372 ± 17.346	0.162
AST (U/L)	24.349 ± 23.986	23.537 ± 27.025	22.772 ± 9.072	26.572 ± 29.711	0.636
Total bilirubin (umol/L)	11.156 ± 5.117	10.571 ± 4.690	11.575 ± 5.298	11.332 ± 5.360	0.524
Albumin (g/L)	43.287 ± 3.437	42.356 ± 3.122	43.683 ± 2.960	43.788 ± 3.956	0.035
LVEF (%)	61.937 ± 5.574	61.924 ± 4.257	62.569 ± 6.021	61.385 ± 6.204	0.503
Cardiovascular medical therapy					
Statin, *n* (%)	170 (86.735)	58 (87.879)	53 (86.885)	59 (85.507)	0.920
ACEI/ARB, *n* (%)	88 (44.898)	37 (56.061)	22 (36.066)	29 (42.029)	0.065
CCB, *n* (%)	82 (41.837)	26 (39.394)	30 (49.180)	26 (37.681)	0.367
Beta blocker, *n* (%)	96 (48.980)	34 (51.515)	30 (49.180)	32 (46.377)	0.836
Aspirin, *n* (%)	140 (71.429)	46 (69.697)	48 (78.689)	46 (66.667)	0.295
Clopidogrel, *n* (%)	97 (49.490)	33 (50.000)	28 (45.902)	36 (52.174)	0.771

CCS, chronic coronary syndrome; CMD, coronary microvascular dysfunction; SBP, systolic blood pressure; DBP, diastolic blood pressure; BMI, body mass index; CKD, chronic kidney disease; PCI, percutaneous coronary intervention; caFFR, coronary angiography-derived fractional fow reserve; caIMR, coronary angiography-derived index of microcirculatory resistance; WBC, white blood cell; TC, total cholesterol; HDL-C, high-density lipoprotein-cholesterol; LDL-C, low-density lipoprotein-cholesterol; TG, triglyceride; SHR, Stress hyperglycemia ratio; FPG, fasting plasma glucose; HbA1c, hemoglobin A1c; Hb, hemoglobin; CRP, C-reactive protein; SCr, serum creatine; AST, aspartate transaminase; LVEF, left ventricular ejection fraction; ACEI/ARB, angiotensin-converting-enzyme inhibitor/angiotensin receptor blocker; CCB, calcium channel blocker.

### Association between the SHR and CMD in CCS patients

As shown in Table 2, the univariable logistic regression analysis identified SHR as being significantly associated with a 7.379-fold increased risk of CMD (OR: 7.379; 95% CI: 1.305–41.725; *P* = 0.024), along with other factors such as BMI, TC, LDL-C, TG, FBG, and the history of hyperlipidemia, PCI, 3-vessel disease, and clopidogrel use. After adjusting for these potential confounders, SHR still proved to be a strong independent risk factor for CMD in the multivariable logistic regression analysis (adjusted OR: 8.955; 95% CI: 1.318–60.825; *P* = 0.025).

**Table 2:** Logistic regression analysis between CMD and clinical risk factors.

Variables	Univariate analysis OR (95% CI)	*P* value	Multivariate analysis OR (95% CI)	*P* value
Age	0.994 (0.973–1.016)	0.601		
Male	0.924 (0.614–1.392)	0.706		
BMI	1.120 (1.047–1.197)	<0.001	1.147 (1.064–1.236)	<0.001
WBC	0.981 (0.871–1.106)	0.756		
TC	1.337 (1.081–1.653)	0.007	1.160 (0.701–1.918)	0.564
HDL-C	0.637 (0.303–1.343)	0.236		
LDL-C	1.365 (1.073–1.737)	0.011	1.073 (0.617–1.865)	0.803
TG	1.198 (1.005–1.427)	0.044	0.907 (0.737–1.117)	0.360
FPG	1.155 (1.015–1.315)	0.029		
Hb1Ac	1.068 (0.900–1.268)	0.453		
cTnT	1.126 (0.700–1.812)	0.625		
Hb	1.008 (0.995–1.022)	0.234		
CRP	1.002 (0.972–1.033)	0.881		
SCr	1.000 (0.991–1.010)	0.931		
AST	1.005 (0.993–1.018)	0.399		
Total bilirubin	0.999 (0.962–1.038)	0.977		
LVEF	1.003 (0.969–1.038)	0.869		
Diabetes	1.303 (0.853–1.989)	0.221		
Hyperlipidemia	2.289 (1.450–3.612)	<0.001	2.271 (1.253–4.117)	0.007
Hypertension	0.972 (0.639–1.477)	0.892		
Heart failure	0.928 (0.129–6.656)	0.941		
Atrial fibrillation	0.606 (0.242–1.519)	0.286		
Stroke	0.958 (0.554–1.658)	0.879		
CKD	1.749 (0.785–3.897)	0.171		
Smoke	1.041 (0.635–1.705)	0.874		
PCI	0.572 (0.380–0.861)	0.007		
1-vessel disease	0.777 (0.496–1.217)	0.270		
2-vessel disease	0.785 (0.482–1.278)	0.330		
3-vessel disease	0.545 (0.303–0.983)	0.044		
Statin, n(%)	0.670 (0.350–1.280)	0.225		
ACEI/ARB, n(%)	0.880 (0.587–1.318)	0.534		
CCB, n (%)	1.161 (0.769–1.752)	0.477		
Beta blocker, n(%)	0.971 (0.649–1.452)	0.884		
Aspirin, n(%)	1.047 (0.671–1.631)	0.841		
Clopidogrel, n(%)	0.527 (0.349–0.797)	0.002	0.526 (0.338–0.817)	0.004
SHR as continuous	7.379 (1.305–41.725)	0.024	8.955 (1.318–60.825)	0.025

HR, hazard ratio, CI, confidence interval; MACE, major adverse cardiovascular event; BMI, body mass index; HR, heart rate; TC, total cholesterol; HDL-C, high-density lipoprotein-cholesterol; LDL-C, low-density lipoprotein-cholesterol; TG, triglyceride; FPG, fasting plasma glucose; HbA1c, hemoglobin A1c; Hb, hemoglobin; CRP, C-reactive protein; SCr, serum creatine; AST, aspartate aminotransferase; LVEF, left ventricular ejection fraction; CKD, chronic kidney disease; PCI, percutaneous coronary intervention; ACEI/ARB, angiotensin-converting-enzyme inhibitor/angiotensin receptor blocker; CCB, calcium channel blocker; SHR, Stress hyperglycemia ratio.

### Clinical outcome

As summarized in Table 3, 53 CMD patients experienced composite MACE during the 32.9-month follow-up period, accounting for 27.0% of the cohort, including 2 cases of cardiac death (2.0%), 14 ischemia-driven revascularizations (7.1%), 4 nonfatal MIs (2.0%), 16 instances of heart failure (8.1%), and 15 nonfatal strokes (7.6%). Patients in the T3 group had a higher prevalence of MACE compared to those in the T1 and T2 groups (16.667% vs. 26.230% vs. 37.681%, respectively; *P* = 0.023).

**Table 3:** Clinical outcomes of the CMD according to SHR tertile.

Clinical outcome	T1 (*N* = 66)	T2 (*N* = 61)	T3 (*N* = 69)	*P* value
MACE	11 (16.667)	16 (26.230)	26 (37.681)	0.023
Cardiac death	1 (1.515)	2 (3.279)	1 (1.449)	0.690
Ischemia-driven revascularization	4 (6.061)	4 (6.557)	6 (8.696)	0.834
Nonfatal MI	1 (1.515)	1 (1.639)	2 (2.899)	1.000
Heart failure	3 (4.545)	4 (6.557)	9 (13.043)	0.169
Nonfatal stroke	2 (3.030)	5 (8.197)	8 (11.594)	0.171

SHR, Stress hyperglycemia ratio, T1, SHR Tertile 1, T2, SHR Tertile 2, T3, SHR Tertile 3, MACE, major adverse cardiovascular event, MI, myocardial infarction.

**Figure 3:**
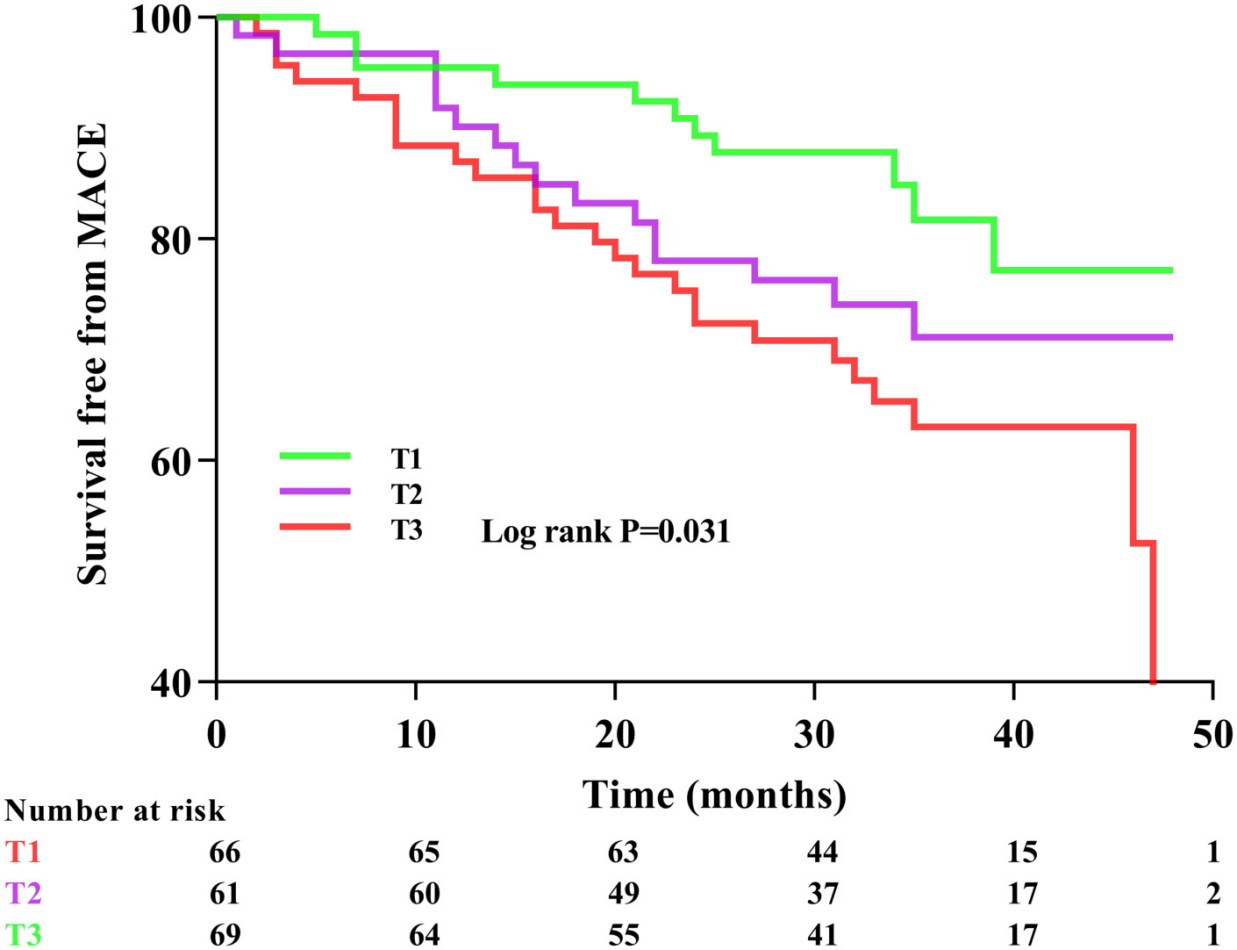
Kaplan–meier survival curve for MACE in CMD patients with CCS according to SHR tertiles. MACE, major adverse cardiovascular events; CMD, coronary microvascular dysfunction; CCS, chronic coronary syndrome; SHR, stress hyperglycemia ratio; T1, SHR Tertile 1; T2, SHR Tertile 2; T3, SHR Tertile 3.

**Figure 4:**
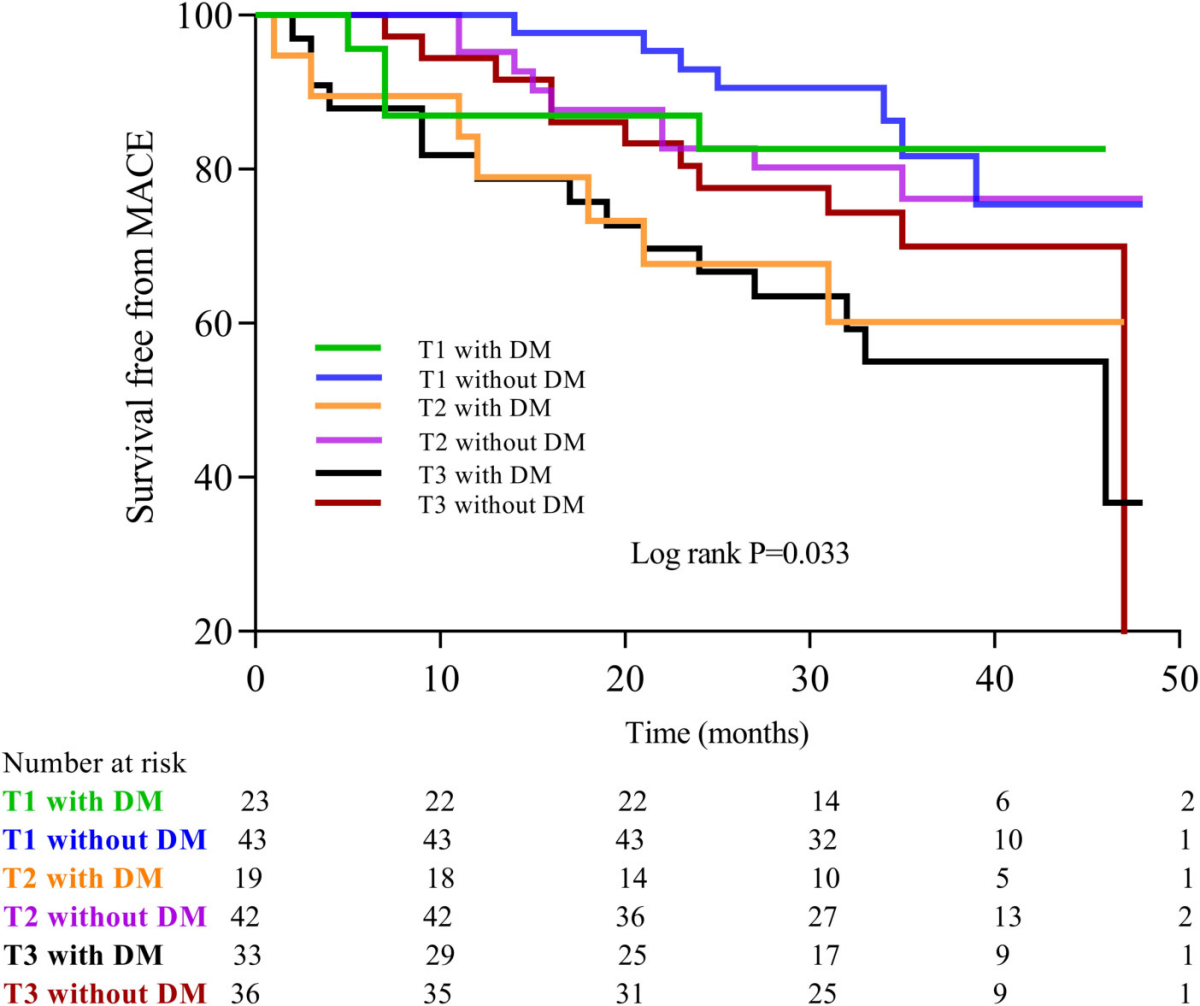
Survival free from MACE based on the SHR tertiles and diabetes status. T1, SHR Tertile 1; T2, SHR Tertile 2; T3, SHR Tertile 3; MACE major adverse cardiovascular event; DM, diabetes mellitus.

To evaluate overall survival, Kaplan–Meier curves were compared using the log-rank test, as shown in Figure 3. The analysis indicated that patients in the T3 group had a significantly higher risk of MACE than those in the other groups (log-rank *P* = 0.031). For a more detailed analysis, the study population was further divided into six subgroups based on SHR tertiles and diabetes mellitus (DM) status: T1 with and without DM, T2 with and without DM, and T3 with and without DM. Results demonstrated that patients in the T3 group with DM had the highest risk of MACE compared to other groups (log-rank *P* = 0.033) (Figure 4).

### Prognostic value of SHR

The results of univariate and multivariate Cox regression analyses for MACE in CMD patients are presented in Table 4. In the univariate Cox regression model, the high SHR group (T3) was identified as a predictive risk factor, associated with a 2.486-fold increased risk of MACE (HR: 2.486; 95% CI: 1.228–5.033; *P* = 0.011). Other significant predictors included FBG, HbA1c, cTnT, AST, LVEF, and a history of DM and stroke (all *P* < 0.05). After adjusting for widely recognized potential confounders, the multivariate Cox regression analysis showed that the T3 group remained significantly associated with a 2.325-fold increased incidence of MACE (HR: 2.325; 95% CI: 1.228–5.033; *P* = 0.011). In contrast, SHR did not show significant prognostic value for survival in the non-CMD patient group (*P* > 0.05) (Supplementary Table 2).

**Table 4:** Cox regression analysis for MACE of the CMD patients.

Variables	Univariate analysis HR (95% CI)	*P* value	Multivariate analysis HR (95% CI)	*P* value
Age	1.025 (0.995–1.055)	0.104		
Male	0.975 (0.567–1.676)	0.927		
BMI	1.015 (0.934–1.102)	0.730		
WBC	1.004 (0.847–1.190)	0.961		
TC	1.104 (0.860–1.418)	0.436		
HDL-C	0.849 (0.321–2.245)	0.741		
LDL-C	1.064 (0.793–1.427)	0.681		
TG	1.097 (0.946–1.272)	0.219		
FPG	1.160 (1.064–1.264)	0.001		
Hb1Ac	1.253 (1.025–1.531)	0.027		
cTnT	1.854 (1.356–2.536)	<0.001	0.932 (0.607–1.432)	0.748
Hb	0.996 (0.981–1.011)	0.613		
CRP	0.994 (0.956–1.034)	0.768		
SCr	1.012 (1.000–1.025)	0.053	1.012 (0.997–1.027)	0.111
AST	1.010 (1.003–1.016)	0.003	1.013 (1.004–1.022)	0.006
Total bilirubin	1.003 (0.948–1.062)	0.907		
LVEF	0.913 (0.880–0.947)	<0.001	0.919 (0.880–0.959)	<0.001
Diabetes	1.829 (1.066–3.136)	0.028	1.383 (0.775–2.471)	0.286
Hyperlipidemia	1.243 (0.718–2.153)	0.437		
Hypertension	1.335 (0.749–2.382)	0.327		
Heart failure	2.946 (0.405–21.425)	0.286		
Atrial fibrillation	2.371 (0.852–6.602)	0.098		
Stroke	1.996 (1.083–3.680)	0.027	2.120 (1.114–4.034)	0.022
CKD	2.056 (0.964–4.385)	0.062		
Smoke	1.164 (0.620–2.184)	0.636		
PCI	0.971 (0.565–1.671)	0.916		
1-vessel disease	0.865 (0.454–1.647)	0.659		
2-vessel disease	1.060 (0.545–2.061)	0.864		
3-vessel disease	1.443 (0.651–3.200)	0.367		
Statin, n(%)	1.210 (0.517–2.831)	0.661		
ACEI/ARB, n(%)	1.452 (0.846–2.491)	0.176		
CCB, n (%)	0.961 (0.555–1.664)	0.887		
Beta blocker, n(%)	0.810 (0.471–1.392)	0.446		
Aspirin, n(%)	0.607 (0.348–1.059)	0.079		
Clopidogrel, n(%)	1.583 (0.911–2.751)	0.103		
SHR as continuous	8.884 (1.626–48.547)	0.012	3.944 (0.693–22.457)	0.122
SHR tertile				
T1	Reference			
T2	1.688 (0.783–3.641)	0.182	1.759 (0.795–3.889)	0.163
T3	2.486 (1.228–5.033)	0.011	2.325 (1.089–4.964)	0.029

HR, hazard ratio; CI, confidence interval; MACE, major adverse cardiovascular event; BMI, body mass index; HR, heart rate; TC, total cholesterol; HDL-C, high-density lipoprotein-cholesterol; LDL-C, low-density lipoprotein-cholesterol; TG, triglyceride; FPG, fasting plasma glucose; HbA1c, hemoglobin A1c; Hb, hemoglobin; CRP, C-reactive protein; SCr, serum creatine; AST, aspartate aminotransferase; LVEF, left ventricular ejection fraction; CKD, chronic kidney disease; PCI, percutaneous coronary intervention, ACEI/ARB, angiotensin-converting-enzyme inhibitor/angiotensin receptor blocker; CCB, calcium channel blocker; SHR, Stress hyperglycemia ratio; T1, SHR Tertile 1; T2, SHR Tertile 2; T3, SHR Tertile 3.

### Non-linear correlation between the SHR and MACE

**Figure 5:**
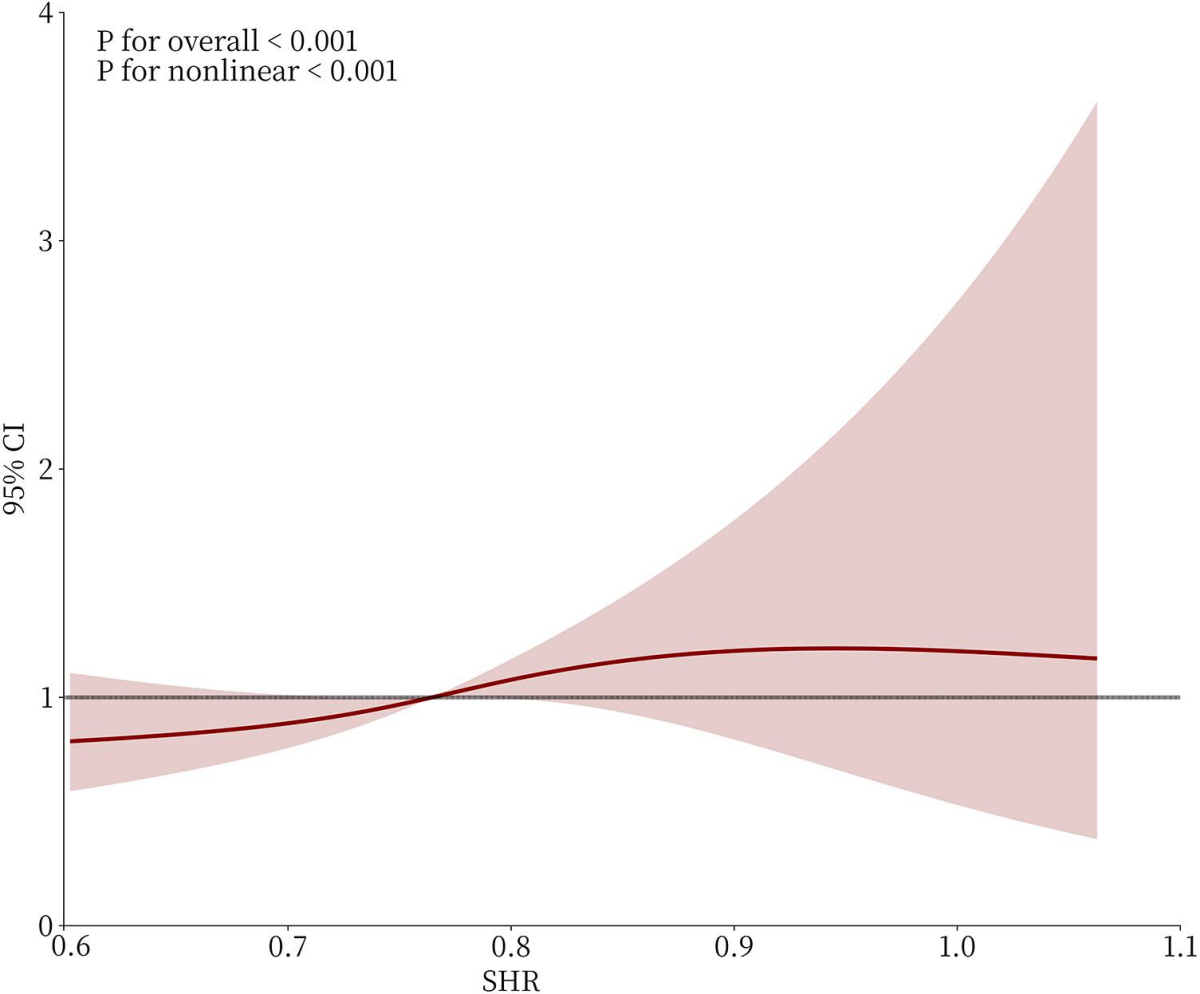
Multivariable adjusted spline curves for associations of the SHR with MACE in CCS patients with CMD. Hazard ratios adjusted for age (as a continuous variable), sex, BMI (as a continuous variable), SCr (as a continuous variable), CTnT (as a continuous variable), LVEF (as a continuous variable), history of DM, hyperlipidemia and nicotine exposure. The solid line and red area represent the estimated values and their corresponding 95% CI. SHR, stress hyperglycemia ratio; HR, hazard ratio; CI, confidence interval.

An adjusted smoothed restricted cubic spline curve, derived from Cox proportional hazards regression analysis, investigated the association between SHR and MACE in the CMD population. The analysis revealed a non-linear association between SHR and MACE after adjusting for factors such as age, sex, BMI, smoking status, presence of DM, hyperlipidemia, and laboratory parameters including SCr, cTnT, and LVEF (P for overall < 0.001, P for non-linear < 0.001) (Figure 5). The risk of MACE remained relatively stable at lower SHR values and increased progressively when SHR exceeded approximately 0.78–0.80. Notably, wide confidence intervals at higher SHR values (>1.0) were observed due to sparse data in this range and these estimates should be interpreted with caution.

### Subgroups analysis

**Figure 6:**
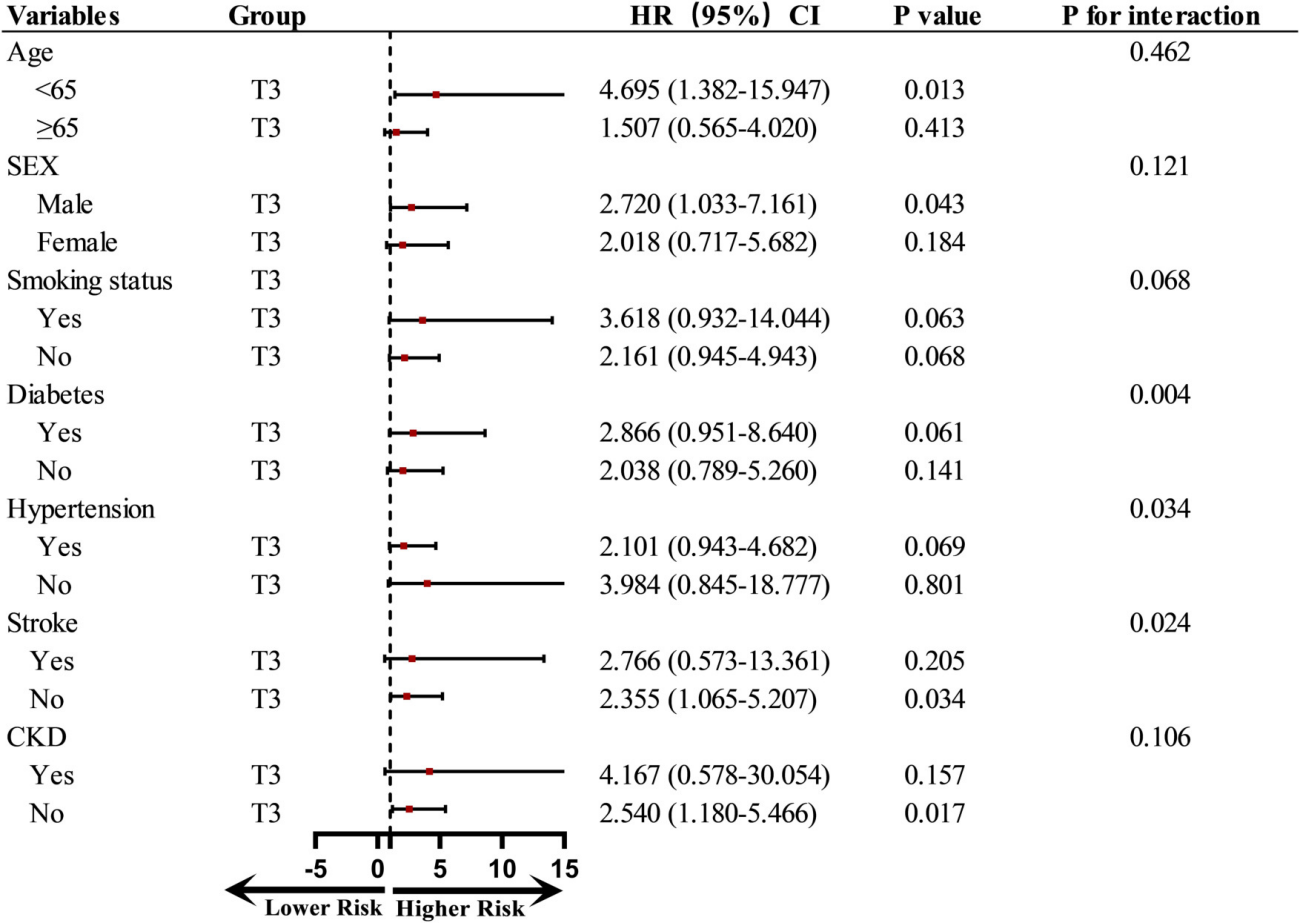
Forest plot of the stratified analyses of the correlations between the SHR and MACE. CKD, chronic kidney disease; HR, hazard ratio; CI, confidence interval.

Stratification and interaction analyses were conducted for factors such as age, sex, smoking status, and history of DM, hypertension, stroke, and CKD to evaluate their potential influence on the relationship between SHR and the risk of MACE (Figure 6). The results revealed significant interactions between SHR and the DM, hypertension, and stroke subgroups (all P for interaction < 0.05). Nominal interactions were observed for diabetes, hypertension, and stroke, but these did not retain statistical significance after correction for multiple testing and should be interpreted with caution given the wide confidence intervals. Although no significant interaction was observed in some subgroups, SHR remained a significant hazard for MACE incidence among males and patients under 65 years of age (HR: 4.695, 95% CI: 1.382–15.947, *P* = 0.013; HR: 2.720, 95% CI: 1.033–7.161, *P* = 0.043, respectively).

## Discussion

To the best of our knowledge, this study is the first to assess the prognostic significance of SHR in relation to CMD in patients with CCS. The key findings of this investigation are as follows: (1) SHR values are significantly higher in CMD patients compared to those without CMD; (2) SHR is an independent risk factor for CMD in CCS patients and is associated with a poorer prognosis of MACE in CMD patients with CCS, demonstrating a non-linear relationship with HR after adjusting for relevant confounding variables; (3) Higher SHR values are significantly correlated with an increased incidence of MACE in patients with DM and stroke, and in those without hypertension, compared to lower SHR values. This study underscores the importance of SHR as a critical index that may help identify CMD patients with CCS at elevated risk of MACE, thereby facilitating targeted screening and more intensive medical interventions.

CCS is a long-term, stable condition characterized by progressive development that often results in severe outcomes and high mortality rates (1, 3). While traditionally attributed to obstructive atherothrombotic disease, a subset of CCS patients experience myocardial ischemia and angina pectoris without detectable abnormalities on CAG. Increasing attention has been given to CMD, which involves structural and functional abnormalities in the coronary microcirculation, impairing blood flow and leading to myocardial ischemia and angina (3, 5). Hyperglycemia-induced oxidative stress and inflammation have been identified as key contributors to CMD, accelerating microvascular aging and dysfunction (4, 13). CMD has been implicated in a range of cardiovascular and systemic conditions, reshaping diagnostic and therapeutic approaches. For example, CMD has been linked to psoriasis severity (13), cancer risk in non-obstructive coronary artery disease (26), and osteoporosis in women (27). Beyond invasive assessment, non-invasive imaging modalities—including stress echocardiography, cardiac magnetic resonance, and positron emission tomography—play an increasingly important role in the comprehensive evaluation of CMD, as comprehensively reviewed by Polimeni et al. (28). These techniques complement invasive approaches such as caIMR by enabling broader patient screening, serial monitoring, and assessment of treatment response without the need for cardiac catheterization. The caIMR has emerged as a reliable, non-invasive metric for assessing CMD with high diagnostic accuracy, eliminating the need for specialized wires or hyperemic agents (21, 29). Studies have associated elevated caIMR with adverse cardiac events in STEMI (30), in-hospital complications in Takotsubo syndrome (31), and increased cardiac death and heart failure-related readmissions in patients with INOCA (29). Furthermore, caIMR has demonstrated prognostic value in predicting MACE in patients with MINOCA, HFpEF, diabetes, or obesity in the context of CCS (10, 32, 33). In our study, we found that 51.7% of CCS patients had CMD, further emphasizing the widespread prevalence of this condition in the CCS population. These findings highlight the critical importance of early detection and diagnosis of CMD and the urgent need for effective risk stratification to improve the typically poor prognosis associated with CCS.

Stress-induced hyperglycemia is strongly associated with CMD by promoting endothelial dysfunction through the production of reactive oxygen species and reducing nitric oxide bioavailability in an inflammatory state (34). It also exacerbates cardiovascular diseases (CVD) through oxidative stress, inflammatory responses, impaired platelet NO responsiveness, and microvascular obstruction. These effects suggest a potential link between Stress-induced hyperglycemia and CMD severity. Stress-induced hyperglycemia, a transient increase in blood glucose upon hospital admission, reflects emergency stress or inadequate glycemic control and is associated with adverse outcomes and worsening cardiovascular diseases CVD (16, 35, 36). Recognizing the limitations of measuring stress-induced hyperglycemia alone, Roberts et al. introduced SHR as a novel biomarker, providing a cost-effective and reliable means to identify true stress-induced hyperglycemia (17). SHR has been extensively studied in cardiovascular conditions such as INOCA, MINOCA, and HFpEF (18, 19, 37), indicating its broader relevance in cardiovascular diseases CVD risk stratification. The identification of reliable circulating biomarkers in CMD carries important therapeutic implications beyond risk stratification. As comprehensively reviewed by Quarta et al. (38), biomarkers associated with endothelial dysfunction, inflammation, and oxidative stress—detectable through routine blood tests—may assist not only in diagnosis but also in guiding personalized treatment strategies and monitoring therapeutic response. In the context of our findings, SHR—as a marker of acute hyperglycemia reflecting the interplay between stress-induced metabolic derangement and chronic glycemic control—suggests several potential therapeutic avenues. First, strict glycemic control during acute illness episodes or procedural interventions may attenuate stress-induced hyperglycemia and its deleterious effects on microvascular function. Second, agents with pleiotropic effects on both glycemic control and microvascular health, such as sodium-glucose cotransporter-2 inhibitors and glucagon-like peptide-1 receptor agonists, may be particularly beneficial in CMD patients with elevated SHR. Third, the non-linear relationship between SHR and outcomes suggests that individualized glycemic targets, rather than one-size-fits-all approaches, may be necessary. Quarta et al. (38) further emphasize that biomarker-guided therapy in CMD should consider the complex interplay between metabolic, inflammatory, and hemodynamic factors. Future randomized trials should evaluate whether SHR-guided treatment strategies—such as intensified glucose-lowering therapy in patients with elevated SHR—can improve clinical outcomes in this high-risk population. The integration of SHR with other emerging biomarkers may ultimately enable a multi-marker approach to personalize CMD management.

### Strengths and limitations

A significant strength of this study is that it is the first to assess the role of SHR in predicting adverse clinical outcomes in CMD patients with CCS. The findings provide valuable insights that can assist physicians in closely monitoring high-risk patients, intensifying goal-directed medical treatment, effectively managing risk factors, and ultimately improving the quality of life for this patient group.

However, several potential limitations should be acknowledged. First, this is a single-center, retrospective observational study with a relatively small sample size. Second, the caIMR technique, which is operator-dependent, may limit its accuracy when compared to invasive IMR, the gold standard for diagnosing CMD. Third, our study did not include dynamic monitoring of fluctuations in stress hyperglycemia; only ABG and HbA1c levels were measured, thus failing to capture the long-term variability of this condition. Although we adjusted for many confounding factors, unmeasured variables could still have influenced the results. Furthermore, since this study was conducted in a Chinese population, the findings may not be directly generalizable to other ethnic groups, which warrants further investigation in diverse populations to confirm the applicability of these results.

## Conclusion

Higher SHR values are linked to poorer prognostic outcomes and serve as a predictor of MACE in CMD patients with CCS. These findings indicate that SHR may serve as a valuable tool for early risk stratification and prevention in this patient group.

## Data Availability

The raw data supporting the conclusions of this article will be made available by the authors, without undue reservation.
