# Supplementary material for: Prognostic significance of stress hyperglycemia ratio in coronary microvascular dysfunction among chronic coronary syndrome patients
Source: Front Cardiovasc Med. 2026 Jun 4;13:1740092. doi: 10.3389/fcvm.2026.1740092 (PMC13275362; doi:10.3389/fcvm.2026.1740092)
Supplement: Supplementary file 1 [file Datasheet1.doc]

**Supplementary Table 1.** Clinical characteristics of CCS patients stratified by CMD status

|  | Total patients  (N=379) | CMD  (N=196) | non-CMD  (N=183) | P value |
| --- | --- | --- | --- | --- |
| General characteristics |  |  |  |  |
| Age (years) | 63.707 ±9.374 | 63.464 ±9.133 | 63.967 ±9.645 | 0.602 |
| Male, n (%) | 222 (58.575) | 113 (57.653) | 109 (59.563) | 0.706 |
| SBP (mmHg) | 135.099 ±58.919 | 138.759 ±78.797 | 131.012 ±20.010 | 0.212 |
| DBP (mmHg) | 78.434 ±12.959 | 80.136 ±12.742 | 76.532 ±12.971 | 0.008 |
| Heart rate (bpm) | 76.662 ±11.307 | 77.158 ±11.400 | 76.131 ±11.213 | 0.378 |
| BMI (kg/m^2^) | 24.227 ±3.477 | 23.765 ±3.880 | 25.151 ±2.968 | 0.607 |
| Comorbidities |  |  |  |  |
| Diabetes, n (%) | 134 (35.356) | 75 (38.265) | 59 (32.240) | 0.220 |
| Hyperlipidemia, n (%) | 114 (30.079) | 75 (38.265) | 39 (21.311) | <0.001 |
| Hypertension, n (%) | 241 (63.588) | 124 (63.265) | 117 (63.934) | 0.892 |
| Heart failure, n (%) | 4 (1.058) | 2 (1.020) | 2 (1.099) | 1.000 |
| Atrial fibrillation, n (%) | 20 (5.277) | 8 (4.082) | 12 (6.557) | 0.281 |
| Stroke, n (%) | 61 (16.095) | 31 (15.816) | 30 (16.393) | 0.879 |
| CKD, n (%) | 28 (7.388) | 18 (9.184) | 10 (5.464) | 0.167 |
| Smoke, n (%) | 80 (21.108) | 42 (21.429) | 38 (20.765) | 0.874 |
| PCI, n (%) | 205 (54.090) | 93 (47.449) | 112 (61.202) | 0.007 |
| 1-vessel disease | 106 (27.968) | 50 (25.510) | 56 (30.601) | 0.270 |
| 2-vessel disease | 83 (21.900) | 39 (19.898) | 44 (24.044) | 0.329 |
| 3-vessel disease | 54 (14.248) | 21 (10.714) | 33 (18.033) | 0.042 |
| Coronary physiological values |  |  |  |  |
| caFFR | 0.929 ±0.055 | 0.949 ±0.026 | 0.907 ±0.069 | <0.001 |
| caIMR | 28.521 ±11.433 | 36.759 ±9.895 | 19.697 ±3.901 | <0.001 |
| Laboratory parameters |  |  |  |  |
| WBC (10^9/L) | 6.526 ±1.743 | 6.499 ±1.657 | 6.556 ±1.838 | 0.757 |
| TC (mmol/L) | 3.800 ±1.001 | 3.935 ±1.063 | 3.653 ±0.911 | 0.006 |
| HDL-C (mmol/L) | 1.094 ±0.275 | 1.078 ±0.285 | 1.112 ±0.264 | 0.236 |
| LDL-C (mmol/L) | 2.062 ±0.878 | 2.175 ±0.924 | 1.941 ±0.811 | 0.010 |
| TG (mmol/L) | 1.763 ±1.407 | 1.912 ±1.536 | 1.603 ±1.240 | 0.034 |
| SHR | 0.766 ±0.121 | 0.780 ±0.136 | 0.751 ±0.103 | 0.022 |
| FPG (mmol/L) | 5.758 ±1.748 | 5.953 ±1.982 | 5.549 ±1.432 | 0.025 |
| Hb1Ac (%) | 6.375 ±1.190 | 6.419 ±1.104 | 6.327 ±1.276 | 0.453 |
| cTnT (ng/mL) | 0.053 ±0.483 | 0.065 ±0.639 | 0.040 ±0.218 | 0.613 |
| Hb (g/L) | 133.874 ±15.372 | 134.792 ±16.502 | 132.890 ±14.041 | 0.230 |
| CRP (mg/dL) | 3.893 ±6.810 | 3.944 ±7.609 | 3.837 ±5.830 | 0.881 |
| SCr (umol/L) | 75.450 ±21.057 | 75.541 ±18.951 | 75.352 ±23.156 | 0.931 |
| AST (U/L) | 23.534 ±18.663 | 24.349 ±23.986 | 22.660 ±10.257 | 0.383 |
| Total bilirubin (umol/L) | 11.163 ±5.427 | 11.156 ±5.117 | 11.172 ±5.762 | 0.977 |
| Albumin (g/L) | 43.262 ±3.737 | 43.287 ±3.437 | 43.235 ±4.032 | 0.894 |
| LVEF (%) | 61.885 ±6.101 | 61.937 ±5.574 | 61.830 ±6.622 | 0.870 |
| Cardiovascular medical therapy |  |  |  |  |
| Statin, n(%) | 336 (88.654) | 170 (86.735) | 166 (90.710) | 0.223 |
| ACEI/ARB, n(%) | 176 (46.438) | 88 (44.898) | 88 (48.087) | 0.534 |
| CCB, n (%) | 152 (40.106) | 82 (41.837) | 70 (38.251) | 0.477 |
| Beta blocker, n(%) | 187 (49.340) | 96 (48.980) | 91 (49.727) | 0.884 |
| Aspirin, n(%) | 269 (70.976) | 140 (71.429) | 129 (70.492) | 0.841 |
| Clopidogrel, n(%) | 216 (56.992) | 97 (49.490) | 119 (65.027) | 0.002 |

*CCS* chronic coronary syndrome*, CMD* coronary microvascular dysfunction*, SBP* systolic blood pressure, *DBP* diastolic blood pressure, *BMI* body mass index*,* CKD chronic kidney disease, *PCI* percutaneous coronary intervention, *caFFR* coronary angiography-derived fractional fow reserve, *caIMR* coronary angiography-derived index of microcirculatory resistance, *WBC* white blood cell, *TC* total cholesterol, *HDL-C* high-density lipoprotein-cholesterol, *LDL-C* low-density lipoprotein-cholesterol, *TG* triglyceride, *SHR* Stress hyperglycemia ratio*, FPG* fasting plasma glucose, *HbA1c* hemoglobin A1c, *Hb* hemoglobin*, CRP* C-reactive protein, *SCr* serum creatine, *AST* aspartate transaminase, *LVEF* left ventricular ejection fraction, *ACEI/ARB* angiotensin-converting-enzyme inhibitor/angiotensin receptor blocker, *CCB* calcium channel blocker

**Supplementary Table 2.** Univariate cox regression analysis for MACE of the non-CMD patients

|  | Univariate analysis  HR (95% CI) | P value |
| --- | --- | --- |
| Age | 1.029 (0.987-1.073) | 0.173 |
| Male | 1.612 (0.701-3.707) | 0.261 |
| BMI | 1.057 (0.931-1.200) | 0.394 |
| WBC | 1.110 (0.912-1.351) | 0.300 |
| TC | 1.226 (0.829-1.815) | 0.308 |
| HDL-C | 0.547 (0.117-2.568) | 0.445 |
| LDL-C | 1.136 (0.723-1.787) | 0.580 |
| TG | 1.028 (0.785-1.347) | 0.838 |
| FPG | 1.063 (0.844-1.339) | 0.605 |
| Hb1Ac | 1.045 (0.799-1.367) | 0.748 |
| cTnT | 0.909 (0.136-6.101) | 0.922 |
| Hb | 1.006 (0.979-1.035) | 0.651 |
| CRP | 1.064 (1.026-1.102) | <0.001 |
| SCr | 1.005 (0.991-1.018) | 0.513 |
| AST | 1.024 (0.990-1.059) | 0.169 |
| Total bilirubin | 0.975 (0.904-1.052) | 0.520 |
| LVEF | 0.895 (0.859-0.933) | <0.001 |
| Diabetes | 1.316 (0.597-2.901) | 0.495 |
| Hyperlipidemia | 1.773 (0.771-4.078) | 0.178 |
| Hypertension | 0.897 (0.407-1.977) | 0.788 |
| Heart failure | 18.514 (4.261-80.453) | <0.001 |
| Atrial fibrillation | 0.557 (0.076-4.114) | 0.567 |
| Stroke | 0.891 (0.307-2.587) | 0.832 |
| CKD | 0.647 (0.088-4.772) | 0.669 |
| Smoke | 1.159 (0.465-2.886) | 0.752 |
| PCI | 5.171 (1.552-17.224) | 0.007 |
| 1-vessel disease | 1.188 (0.529-2.666) | 0.676 |
| 2-vessel disease | 1.184 (0.498-2.817) | 0.702 |
| 3-vessel disease | 2.121 (0.922-4.879) | 0.077 |
| Statin, n(%) | 1.208 (0.285-5.112) | 0.797 |
| ACEI/ARB, n(%) | 2.109 (0.940-4.731) | 0.070 |
| CCB, n (%) | 1.674 (0.776-3.613) | 0.189 |
| Beta blocker, n(%) | 1.386 (0.636-3.021) | 0.411 |
| Aspirin, n(%) | 1.386 (0.636-3.021) | 0.411 |
| Clopidogrel, n(%) | 1.463 (0.615-3.482) | 0.389 |
| SHR as continuous | 1.766 (0.039-79.986) | 0.770 |
| SHR tertile |  |  |
| T1 | Reference |  |
| T2 | 0.443 (0.164-1.197) | 0.108 |
| T3 | 0.865 (0.358-2.087) | 0.746 |

*HR* hazard ratio, *CI* confidence interval, *MACE* major adverse cardiovascular event, *BMI* body mass index, *HR* heart rate, *TC* total cholesterol, *HDL-C* high-density lipoprotein-cholesterol, *LDL-C* low-density lipoprotein-cholesterol, *TG* triglyceride, *FPG* fasting plasma glucose, *HbA1c* hemoglobin A1c, *Hb* hemoglobin, *CRP* C-reactive protein, *SCr* serum creatine, *AST* aspartate aminotransferase, *LVEF* left ventricular ejection fraction, *CKD* chronic kidney disease, *PCI* percutaneous coronary intervention, *ACEI/ARB* angiotensin-converting-enzyme inhibitor/angiotensin receptor blocker, *CCB* calcium channel blocker, *SHR* Stress hyperglycemia ratio, *T1* SHR Tertile 1, *T2* SHR Tertile 2, *T3* SHR Tertile 3
